# Molecular and Clinical Characterization of PD-1 in Breast Cancer Using Large-Scale Transcriptome Data

**DOI:** 10.3389/fimmu.2020.558757

**Published:** 2020-11-17

**Authors:** Qiang Liu, Ran Cheng, Xiangyi Kong, Zhongzhao Wang, Yi Fang, Jing Wang

**Affiliations:** Department of Breast Surgical Oncology, National Cancer Center/National Clinical Research Center for Cancer/Cancer Hospital, Chinese Academy of Medical Sciences and Peking Union Medical College, Beijing, China

**Keywords:** breast cancer, cancer immunotherapy, PD-1, immune response, inflammatory activity

## Abstract

Despite the impressive impact of PD-1 (programmed cell death protein 1)-targeted cancer immunotherapy, a great part of patients with cancer fail to respond. PD-1 impact on immune cells in addition to T cells, and the synergistic role of PD-1 with other immune modulators remain largely unknown. To fill this gap, we systematically investigated PD-1-related transcriptome data and relevant clinical information derived from TCGA (The Cancer Genome Atlas) and METABRIC (Molecular Taxonomy of Breast Cancer International Consortium), which involved a total of 2,994 breast cancer patients. Our results revealed the relationship among PD-1 and major molecular and clinical characteristics in breast cancer. More importantly, we depicted the association landscape between PD-1 and other immune cell populations. Gene ontology analyses and gene set variation analysis (GSVA) of genes correlated with PD-1 revealed that PD-1 was mainly involved in immune responses and inflammatory activities. We also elucidated the association of PD-1 with other immune modulators in pan-cancer level, especially the potential synergistic relationship between PD-1 and other immune checkpoints members in breast cancer. In short, the expression level of PD-1 was bound up with breast cancer malignancy, which could be used as a potential biomarker; PD-1 might manipulate the anti-tumor immune response by impacting not just T cells, and this might vary among different tumor types. Furthermore, PD-1 might synergize with other immune checkpoint members to modulate the immune microenvironment in breast cancer.

## Introduction

Breast cancer is the most frequently diagnosed women cancer all over the world (1). As development of comprehensive treatments, including resection by surgery, chemotherapy, radiotherapy, endocrine therapy, and targeted therapy, many early stage breast cancers can be effectively controlled (2). However, for advanced breast cancers, the existing standard treatments have limited effectiveness because of the aggressiveness of tumors, resistance to treatments, recurrence, and metastasis during or after treatments (3). In the past decade, researches suggested that tumor cells’ immunologic escape of and aberrant human immune surveillance play essential roles in the carcinogenesis, progression, and metastasis of cancers (4), and studies focused on anticancer immune responses have achieved marked success of many malignant tumors in preclinical and clinical trials (5–9), the PD-1 (programmed cell death protein 1) and PD-L1 (programmed cell death-ligand 1) axis has been identified as one of the most encouraging findings in cancer immunotherapy (10).

PD-1 is a receptor that is expressed on the surface of activated T cells, and the PD-L1 and PD-L2 are ligands of PD-1 that are expressed on the surface of antigen-presenting cells (11). The PD-1 and PD-L1 interaction can result in T cells inactivation and ensure that the immune system can be activated at the appropriate time, thus minimize the possibility of chronic autoimmune inflammation. However, tumor cells or non-transformed cells in the tumor microenvironment overexpressing PD-L1, leading to generate an adaptive immune resistance in response to endogenous immune anti-tumor activity (12). The antibody of PD-1/PD-L1 can block the immune escape mediated by PD-1 and PD-L1 interaction and has been approved by the FDA in a fast speed (13). In the first clinical trials of breast cancer, the inhibitors of PD-1/PD-L1 showed promising activity (14, 15). However, many problems still need to be resolved, including the lack of clearly available data in breast cancer, notably regarding PD-1 expression and its prognostic value, and the application of PD-1/PD-L1 inhibitors in combination with other immune checkpoint inhibitors.

Despite the impressive impact of PD-1/PD-L1-targeted cancer immunotherapy, a large proportion of cancer patients fail to respond (16). Furthermore, although the combination of PD-1/PD-L1 blockade with complementary checkpoint inhibitors has achieved some success for some malignant tumors in the preclinical and clinical trials, the impact of PD-1 on immune cells in addition to T cells and the synergistic role of PD-1 with other immune modulators remain mostly unknown (17). In the present study, we systematically investigated the PD-1-related transcriptome profile and revealed its potential role in inducing immune responses and inflammatory activities as well as its potential relationship with immune modulators.

## Methods

### Data Collection

Transcriptome data from TCGA (The Cancer Genome Atlas) were downloaded by GDCRNA tools (access date: February 01, 2020) (18). The edgeR (19) and limma packages (20) available from the Bioconductor project (21) offer a well-developed suite of statistical methods for dealing with this question for RNA-seq data. Raw count data were normalized using TMM implemented in edgeR, and then were transformed by voom in limma, only genes with cpm > 1 in more than half of the samples were kept. Sieved TCGA breast cancer clinical data were kindly provided by Dr. Hai Hu and Dr. Jianfang Liu (Chan Soon-Shiong Institute of Molecular Medicine, Windber). Human epidermal growth factor receptor 2 (HER2) status was recalled using DNA copy number for cases without immunohistochemistry or fluorescence *in situ* hybridization (FISH) status. Standardized survival data of TCGA cohort were downloaded from TCGA-CDR (TCGA Pan-Cancer Clinical Data Resource) (22). The METABRIC (Molecular Taxonomy of Breast Cancer International Consortium) dataset (23) containing 1904 tumor cases was downloaded from the cBioPortal database (http://www.cbioportal.org/) (access date: Feb 01, 2019). A total of 2,994 samples with full clinical characteristics and transcriptome data were used to perform the following data exploration. The detailed clinical characteristics of breast cancer patients from TCGA and METABRIC are listed in **Tables S1** and **S2**, respectively).

### Bioinformatics Analysis

**Figure 1:**
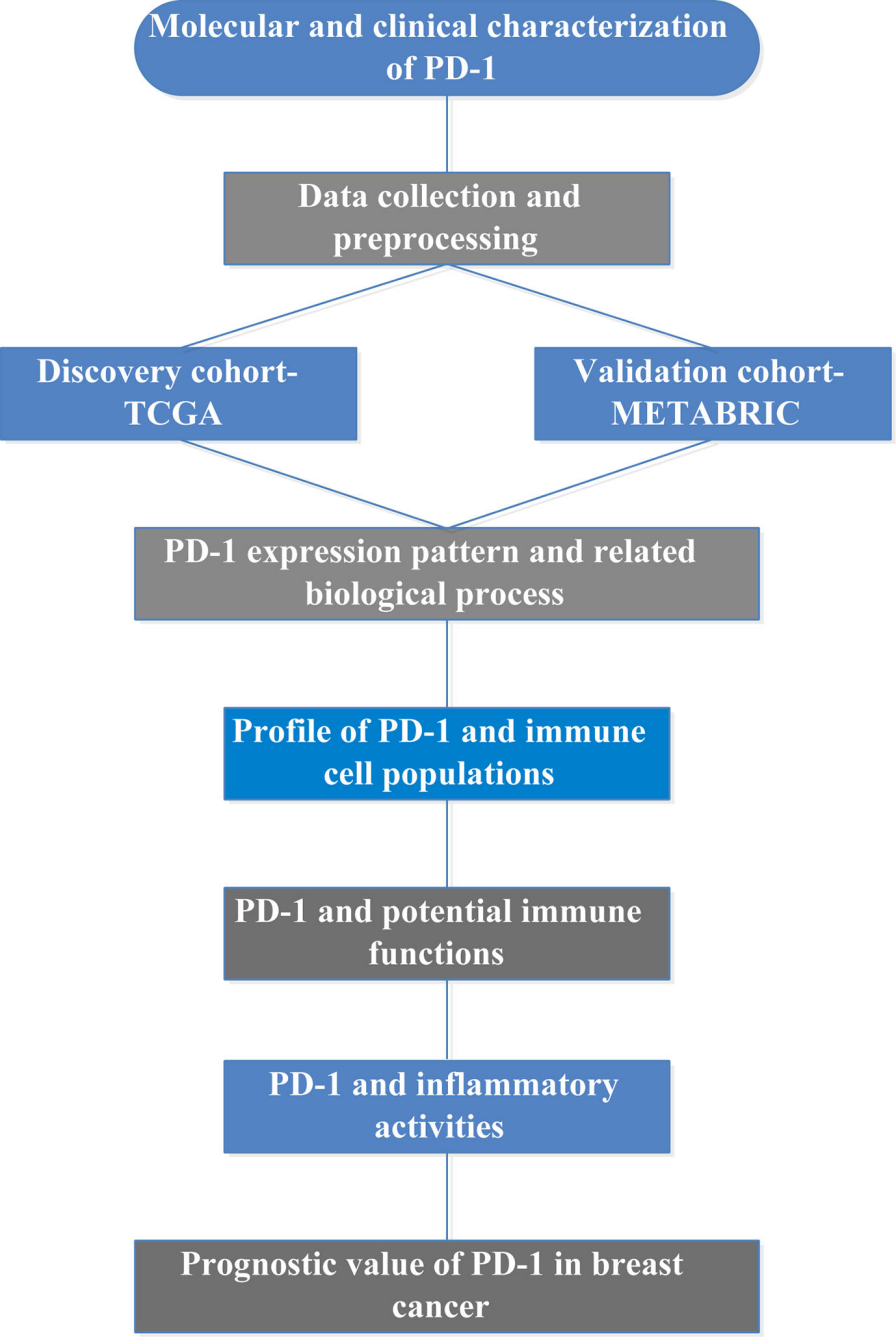
Summary diagram of the present study.

Gene ontology analyses of the genes that correlated with PD-1 were performed using clusterProfiler package (24). Immunologically related genes were collected from The ImmPort (Immunology Database and Analysis Portal) database (https://www.immport.org/home) (25). The absolute abundance of immune cell populations was estimated using Microenvironment Cell Populations-counter algorithm (26). GSVA (Gene Set Variation Analysis) (27) was used to calculate scores of gene sets that correlated with immune functions and inflammatory activities (28). Association of PD-1 and other immune modulators in pan-cancer were depicted through the database of TISIDB (29), which is an integrated repository portal for tumor-immune system interactions. The study summary diagram is shown in **Figure 1**.

### Statistical Analysis

Spearman correlation method was used to estimate the correlations between continuous variables. Student t-test, one-way ANOVA, or Pearson’s Chi-squared test were used to determine any differences in variables between groups. R language was used to perform all statistical tests. The prognostic value of PD-1 was evaluated through Cox proportional hazards model analysis. Several packages including ggplot2 pheatmap, pROC (30), circlize (31), and corrgram (32) were used to perform other statistical calculations and graphical work (33), and P < 0.05 was considered to have the statistically significant difference.

## Results

### PD-1 Expression Pattern in Breast Cancer

**Figure 2:**
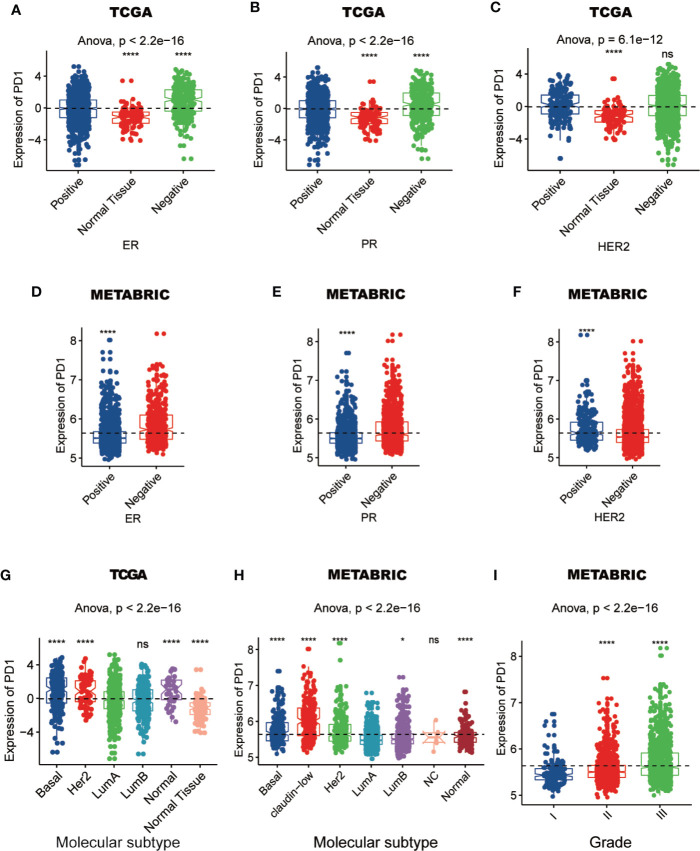
The expression of PD-1 in different ER, PR, and HER2 status **(A–F)**, molecular subtypes **(G, H)**, and grades **(I)** in TCGA or METABRIC cohort. (*: P < 0.05, ****: P < 0.0001, ns: no significant difference).

**Figure 3:**
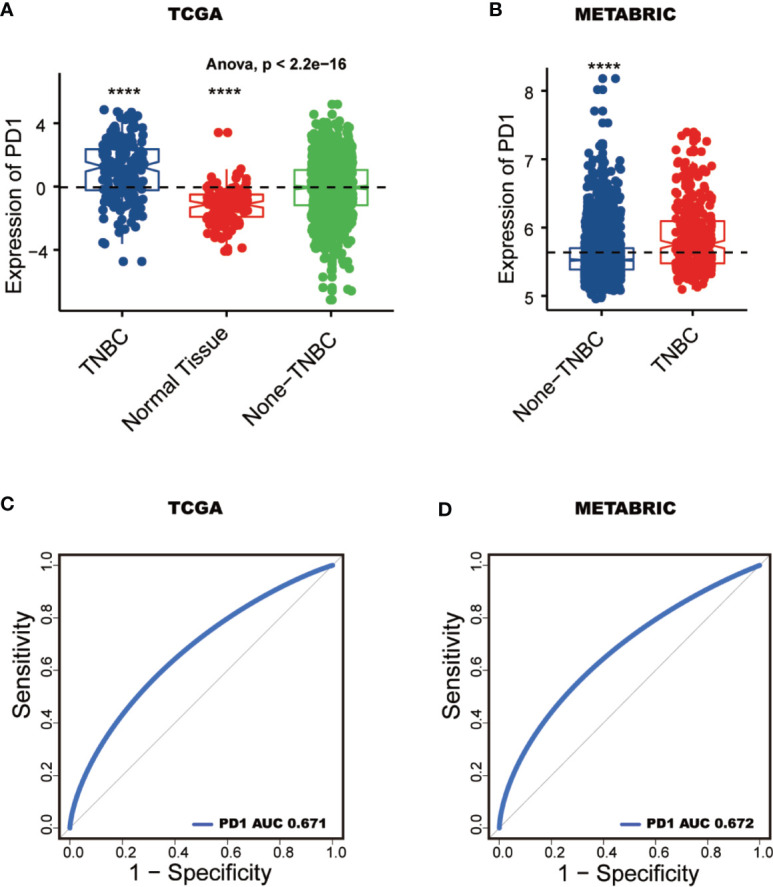
PD-1 serves as a potential biomarker. PD-1 expression pattern between TNBC and none-TNBC tissues in TCGA and METABRIC **(A**, **B)**. ROC curves predict PD-1 as a biomarker of TNBC **(C**, **D)**. (****: P < 0.0001).

To characterize the relationship between PD-1 expression and molecular and clinical features in breast cancer, individuals were dichotomized into high and low groups based on the expression of PD-1 using median cut. We found that PD-1 was asscociated with patient age, American Joint Committee on Cancer (AJCC) stage, tumor grade, estrogen receptor (ER) status, progesterone receptor (PR) status and HER2 status (**Tables 1** and **2**). Next, we further detected that the PD-1 expression was upregulated in tumor tissues, the ER-negative group (ER-) and PR-negative group (PR-) in both TCGA and METABRIC datasets, while upregulation of PD-1 in the HER2-positive group was only observed in the METABRIC dataset (**Figures 2A–F**). Meanwhile, we observed that PD-1 expression was upregulated in the molecular subtypes such as basal-like and HER2-enriched when compared with luminal A, while no difference was found between luminal A and luminal B subtypes. While no claudin-low subtype was found in the TCGA cohort, the other four subtypes were consistently in both the TCGA and METABRIC cohorts (**Figures 2G, H**). Subsequently, we also found that PD-1 expression was enriched in higher grade tumors in the METABRIC dataset (**Figure 2I**). PD-1 overexpression was also oberved in the triple-negative breast cancer (TNBC) subtype and could be as a predictor for TNBC subtype in both TCGA (AUC = 0.671) and METABRIC (AUC = 0.672) databases (**Figures 3A–D**). One potential limitation of this result was that this was a primary result based on only one gene. Future studies focusing on investigating robust biomarkers for TNBC might consider combining PD-1 and other biomarkers together, and comparing the predictive capacity between each other. In summary, these findings suggest that PD-1 expression is enriched in higher malignant breast cancer and might be a potential biomaker in TNBC.

**Table 1:** Association between PD-1 mRNA expression and clinicopathologic characteristics in TCGA cohort.

		Expression		
	Total (n = 1090)	PD-1 high (n = 545)	PD-1 low (n = 545)	P-value
**Age (years)**				
>=55	517 (47.4%)	277 (50.8%)	240 (44.0%)	0.025
<55	573 (52.6%)	268 (49.2%)	305 (56.0%)	
**T stage**				
T1	279 (25.6%)	130 (23.9%)	149 (27.3%)	0.028
T2	631 (57.9%)	330 (60.6%)	301 (55.2%)	
T3	137 (12.6%)	72 (13.2%)	65 (11.9%)	
T4	40 (3.7%)	13 (2.4%)	27 (5.0%)	
Unknown	3 (0.3%)	0 (0%)	3 (0.6%)	
**N stage**				
N0	514 (47.2%)	257 (47.2%)	257 (47.2%)	0.065
N1	360 (33.0%)	177 (32.5%)	183 (33.6%)	
N2	120 (11.0%)	57 (10.5%)	63 (11.6%)	
N3	76 (7.0%)	48 (8.8%)	28 (5.1%)	
Unknown	20 (1.8%)	6 (1.1%)	14 (2.6%)	
**M stage**				
M0	907 (83.2%)	452 (82.9%)	455 (83.5%)	0.061
M1	22 (2.0%)	6 (1.1%)	16 (2.9%)	
Unknown	161 (14.8%)	87 (16.0%)	74 (13.6%)	
**AJCC stage**				
I	181 (16.6%)	82 (15.0%)	99 (18.2%)	0.097
II	621 (57.0%)	319 (58.5%)	302 (55.4%)	
III	250 (22.9%)	131 (24.0%)	119 (21.8%)	
IV	20 (1.8%)	5 (0.9%)	15 (2.8%)	
Unknown	18 (1.7%)	8 (1.5%)	10 (1.8%)	
**ER status**				
Negative	236 (21.7%)	155 (28.4%)	81 (14.9%)	<0.001
Positive	803 (73.7%)	372 (68.3%)	431 (79.1%)	
Unknown	51 (4.7%)	18 (3.3%)	33 (6.1%)	
**PR status**				
Negative	343 (31.5%)	203 (37.2%)	140 (25.7%)	<0.001
Positive	694 (63.7%)	323 (59.3%)	371 (68.1%)	
Unknown	53 (4.9%)	19 (3.5%)	34 (6.2%)	
**HER2 status**				
Negative	895 (82.1%)	449 (82.4%)	446 (81.8%)	0.032
Positive	168 (15.4%)	89 (16.3%)	79 (14.5%)	
Unknown	27 (2.5%)	7 (1.3%)	20 (3.7%)	

**Table 2:** Association between PD-1 mRNA expression and clinicopathologic characteristics in METABRIC cohort.

		Expression		
	Total (n = 1904)	PD-1 high (n = 952)	PD-1 low (n = 952)	P-value
**Age (years)**				
>=55	952 (50.0%)	522 (54.8%)	430 (45.2%)	<0.001
<55	952 (50.0%)	430 (45.2%)	522 (54.8%)	
**Tumor size**				
>=2cm	592 (31.1%)	293 (30.8%)	299 (31.4%)	0.079
<2cm	1292 (67.9%)	644 (67.6%)	648 (68.1%)	
Unknown	20 (1.1%)	15 (1.6%)	5 (0.5%)	
**AJCC stage**				
0	4 (0.2%)	3 (0.3%)	1 (0.1%)	0.039
I	475 (24.9%)	218 (22.9%)	257 (27.0%)	
II	800 (42.0%)	413 (43.4%)	387 (40.7%)	
III	115 (6.0%)	68 (7.1%)	47 (4.9%)	
IV	9 (0.5%)	2 (0.2%)	7 (0.7%)	
Unknown	501 (26.3%)	248 (26.1%)	253 (26.6%)	
**Tumor grade**				
I	165 (8.7%)	50 (5.3%)	115 (12.1%)	<0.001
II	740 (38.9%)	311 (32.7%)	429 (45.1%)	
III	927 (48.7%)	551 (57.9%)	376 (39.5%)	
Unknown	72 (3.8%)	40 (4.2%)	32 (3.4%)	
**ER status**				
Negative	445 (23.4%)	309 (32.5%)	136 (14.3%)	<0.001
Positive	1459 (76.6%)	643 (67.5%)	816 (85.7%)	
**PR status**				
Negative	895 (47.0%)	526 (55.3%)	369 (38.8%)	<0.001
Positive	1009 (53.0%)	426 (44.7%)	583 (61.2%)	
**HER2 status**				
Negative	1668 (87.6%)	800 (84.0%)	868 (91.2%)	<0.001
Positive	236 (12.4%)	152 (16.0%)	84 (8.8%)	

### PD-1 Was Bound Up With Immune Functions in Breast Cancer

**Figure 4:**
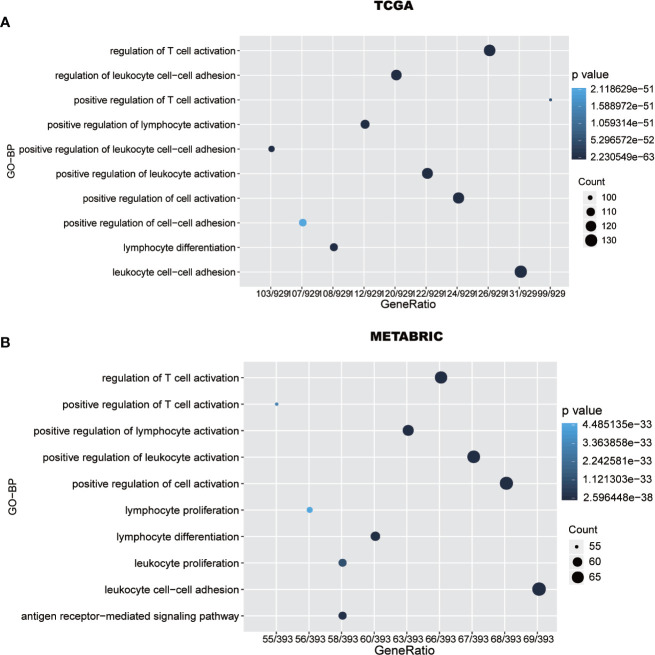
PD-1 is closely related to immune functions in breast cancer. Gene ontology analysis shows that PD-1 is mainly involved in immune response and inflammatory response in TCGA and METABRIC databases **(A**, **B)**.

To further explore PD-1 related biological processes in breast cancer, a total of 1008 genes and 449 genes, seived from TCGA and METABRIC datasets, respectively, have strongly correlation with PD-1 according to Spearman correlation analysis (|R| > 0.4 and P < 0.05). Subsequently, GO (gene ontology) enrichment analyses were performed to investigate PD-1’s potential biological functions. We found that PD-1-related genes were mainly involved in immune-related pathways and inflamatory pathways (**Figure 4A**), including T cell regulation-related biological processes and leukocyte regulation-related pathways. Importantly, these results were also validated in METABRIC datasets (**Figure 4B**).

### PD-1 Related Immune Response

To further investigate PD-1-related immune functions in breast cancer, 4723 immunologically related genes were retrieved from the ImmPort (25). Genes that were most correlated with PD-1 (Spearman |R| > 0.4, P < 0.05) were seived to depict the expression pattern of these immunologically related genes in breast cancer. Subsequently, we found that 508 and 248 immune-related genes in TCGA and METABRIC datasets, respectively, were correlated with PD-1 postively, while only 6 and no immunologically related genes, respectively, were correlated with PD-1 negatively (**Figures S1A, B**).

### The Relationship Between PD-1 Expression and Immune Cell Populations

**Figure 5:**
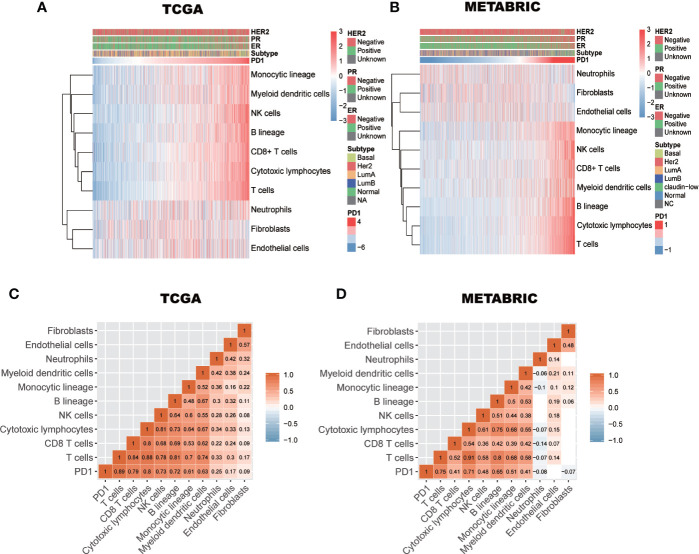
The relationship between PD-1 expression and immune cell populations in TCGA and METABRIC databases **(A**–**D)**. Subtypes denotes breast cancer molecular subtypes including Basal, basal-like; Her2, Her2-enriched; LumA, luminal A; LumB, luminal B.

Previously, Tao Jiang et al. reported PD-1 is a positively correlated with T cells, monocytic lineage cells, and myeloid dendritic cells, but not with cytotoxic lymphocytes, natural killer (NK) cells, or B lineage cells in diffuse glioma (34). In breast cancer, to further clarify the immune manipulative functions of PD-1, the Microenvironment Cell Populations-counter algorithm was used to calculate the absolute abundance of immune cell populations (26). The abundance pattern of these cell populations in breast cancer is depicted in **Figures 5A, B**. We observed that PD-1 expression was strongly correlated with immune cell population scores of T cells, CD8+ T cells, cytotoxic lymphocytes, NK cells, B lineage cells, monocytic lineage cells, and myeloid dendritic cells, but not neutrophils, endothelial cells, or fibroblasts (**Figures 5C, D**). These findings suggest that PD-1 may not just be involved in regualting T cell immunity, other immune cell immunity might also be involved. Furthermore, the immune regulatory pattern of PD-1 may be varied in different tumors.

### Relationship of the Expression of PD-1 and Immune Modulators in Pan-Cancer

To explore the synergistic role of PD-1 and other immune modulators in pan-cancer, we systematically analyzed the correlations between PD-1 expression and three types of immune modulators described in the previous study conducted by Charoentong et al. (35). Interestingly, we found a similar correlation pattern between immune modulators and PD-1 across 30 tumor types in which the majority of immunoinhibitors and immunostimulators were correlated with PD-1 positively (**Figures S2** and **S3**), while only a small number of immunoinhibitors and immunostimulators were negatively correlated with PD-1. More interestingly, we observed that PD-1 was positively correlated with almost all MHC molecules across 30 tumor types (**Figure S4**). These results provide a landscape perspective regarding the correlation of PD-1 with immune modulators, and we could compare this correlation pattern of PD-1 with immune modulators in various tumors. The general PD-1 correlation pattern trends were similar in various tumors. These observations suggest that PD-1 might synergize with other immune modulators in manipulating the anti-tumor immune response.

### PD-1 Is Synergistic With Other Immune Checkpoint Members in Breast Cancer-Induced Immune Response

**Figure 6:**
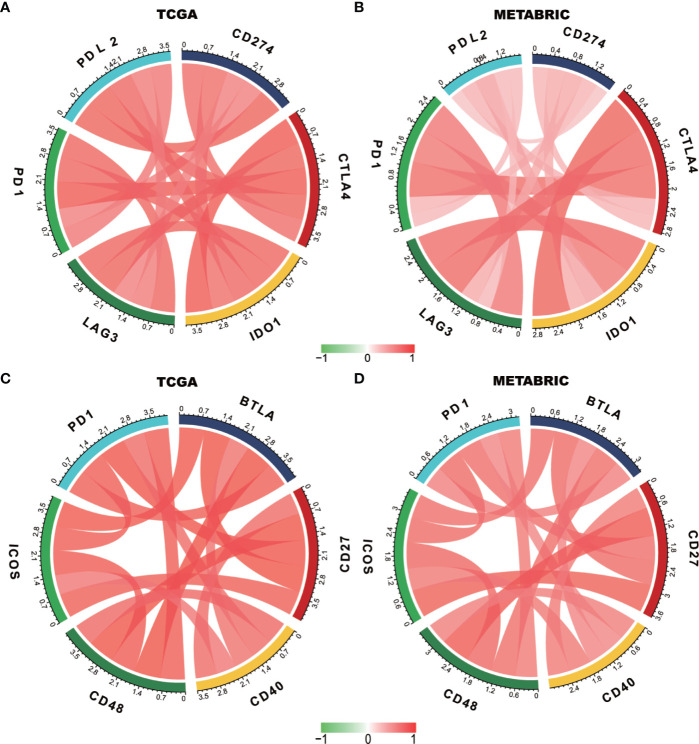
PD-1 expression is correlated with immune checkpoint members in TCGA and METABRIC databases **(A**–**D)**.

We estimated the association of PD-1 with other immune checkpoint members to further explore the synergistic role of PD-1 in breast cancer-induced immune responses (**Figures 6A–D**). The detailed R and p-values of correlations between PD-1 and other checkpoint members are listed in **Tables S3**. Interestingly, we found that PD-1 was strongly correlated with similar checkpoint members including PD-L2, CD274 (PD-L1) CTLA4, IDO1, and LAG3, as well as other checkpoint members including BTLA, ICOS, CD27, CD40, and CD48. These findings revealed that PD-1 might manipulate anti-tumor immune responses through co-regulation with the above mentioned immune checkpoint molecules, thereby lending support to using combination cancer immunotherapy targeting these molecules in future studies.

### Asscociation Between PD-1 and Specific Cell Immune Responses

**Figure 7:**
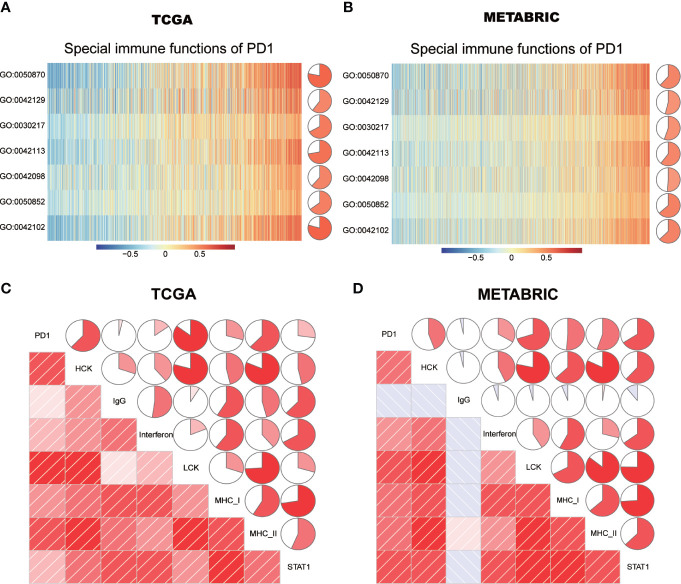
PD-1-related cell immunity and inflammatory activities in breast cancer. The relationship between PD-1 and cell immunity in TCGA and METABRIC datasets **(A**, **B)**. The relationship between PD-1 and inflammatory activities in TCGA and METABRIC datasets **(C**, **D)**. GO:0030217: T cell differentiation; GO:0042098:T cell proliferation; GO:0042102: positive regulation of T cell proliferation; GO:0042113: B cell activation; GO:0042129: regulation of T cell proliferation; GO:0050852: T cell receptor signaling pathway; GO:0050870: positive regulation of T cell activation. The pie denotes the correlation coefficient of PD-1 and GO term.

The specific immune regulatory role of PD-1 in breast cancer remains largely unknown. To further explore the association between PD-1 and specific immune responses, GSVA of gene ontology biological pathways was performed. Consistent with the above results, we found that PD-1 was strongly correlated with both T and B cell immunity (**Figures 7A, B**). PD-1 was positively correlated with T cell proliferation, T cell differentiation, T cell activation and T cell receptor signaling pathways. Meanwhile, we also observed that PD-1 was positively correlated with B cell activation.

### Relationship Between PD-1 and Inflammatory Activities

To further understand the role of PD-1 in mediating inflammatory activities, 104 genes were derived from seven clusters and defined as metagenes using the GSVA algorithm (28), implicating different types of inflammation and immune functions. Detailed information of these metagenes is shown in **Table S4**. We found that LAG3 was positively correlated with MHC-I, MHC-II, LCK, STAT1, HCK, and interferon, but not IgG (**Figures 7C, D**). Among these seven clusters, PD-1 showed the strongest correlation with MHC-II and LCK in both TCGA and METABRIC databases. These results further suggested that PD-1 not only correlated with T cell immunity but also with other immune cells. In summary, these findings indicated that PD-1 has important immune and inflammatory functions in breast cancer.

### Prognostic Value of PD-1 in Breast Cancer

**Figure 8:**
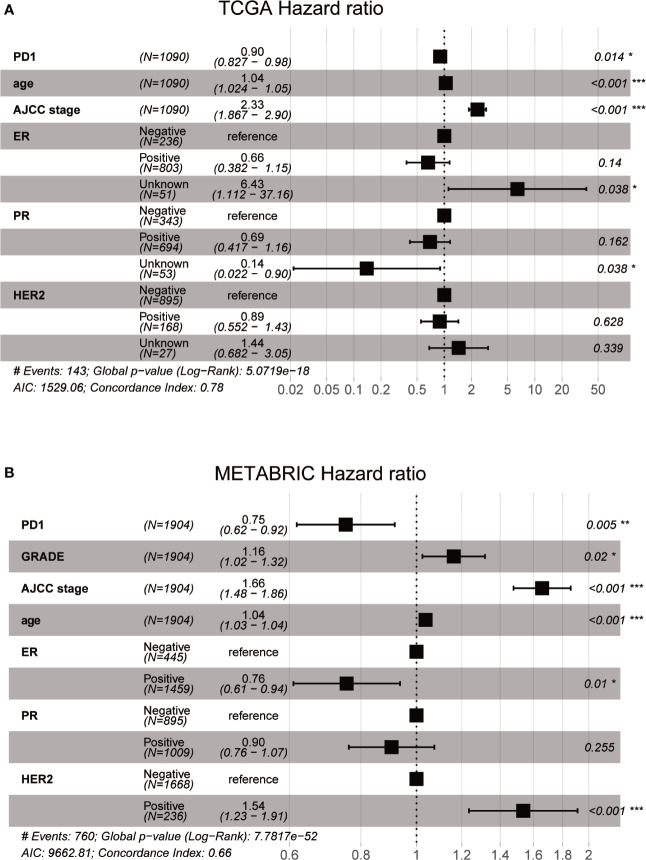
Prognostic value of PD-1 in breast cancer using TCGA and METABRIC databases (**A** and **B**).

To explore the influence on breast cancer survival, we evaluated the prognostic value of PD-1 in both TCGA (n = 1090) and METABRIC (n = 1994) cohorts. Interestingly, our data indicated that PD-1 was an independent prognostic factor in breast cancer on the basis of TCGA cohort multivariate analysis after adjusting for patient age, AJCC stage, ER, PR, and HER2 status (**Figure 8A**). In the METABRIC cohort, the PD-1 expression level was also an independent prognostic indicator for breast cancer after adjusting for tumor grade, AJCC stage, ER, PR, and HER2 status (**Figure 8B**). Despite the fact that PD-1 was upregulated in higher malignant tumors, our results suggest that PD-1 is predictive of good prognosis in breast cancer patients.

## Discussion

The development of immune therapies for solid tumors have promoted clinical advances (36), PD-1/PD-L1 are critical biological suppressors of cytotoxic immune reactions, and the PD-L1 expression is one of the major immunologic escape mechanisms in tumors (37). However, despite the impressive impact of PD-1/PD-L1-targeted cancer immunotherapy, a large proportion of cancer patients fail to respond. To understand why this occurs, we need to have a better understanding of the molecular regulatory mechanisms of PD-1/PD-L1.

Whether PD-1/PL-L1 predicts prognosis in breast cancer patients is currently being debated, and large-scale investigations are still required to further confirm the specific relationship between PD-1/PD-L1 and prognosis of breast cancer (38). PD-1 was found to be significantly associated with better DFS and OS in Ren et al.’s study of only 195 TNBC patients (39). The present study used large-scale transcriptome data to provide additional strong evidence to support the correlation between high PD-1 expression and good prognosis in breast cancer patients. We estimated the association of PD-1 with other checkpoint proteins and found that PD-1 was strongly correlated with similar checkpoint members. Some of these results were consistent with previous studies, while some results have never been reported before. For instance, previous studies reported that LAG-3 and PD-1 were co-expressed on tumor-infiltrating lymphocytes, and blockade of both pathways had synergistic effects on anti-tumor CD8+ T cell stimulation and response (40, 41).

In the present study, one interesting finding was that the immune inhibitors and immune stimulators were positively and concomitantly correlated with PD-1 expression. For primary and secondary resistance to immunotherapy, the etiologies are multifaceted, tumor intrinsic factors, and the complex interplay between cancer and its microenvironment all should to be taken into consideration (42). One possible explanation for immunotherpy resistance might be that there are complex interactions between PD-1 expression, immune inhibitors, and immune stimulators. Hence, the result that immune inhibitors and immune stimulators are positively correlated at the same time in the same sets of patients is reflective of a complex tumor microenvironment. More importantly, these results also suggest that when using immunotherapy, both activation of stimulatory pathways and blockade of inhibitory checkpoints can occur and should therefore be taken into consideration.

It is generally agreed that PD-1 mainly inhibits the activation and immunologic function of T cells (43). We found that PD-1 was not only strongly correlated with T cell immunity, but also with B cell immunity. Tumor-infiltrating B cells with distinct phenotypes and functions, which might play specific roles in the anti-tumor responses (44). However, there is still a lack of direct evidence to support B cells have the immunosuppressive role in human cancers (45). Further study is needed to explore the possible manipulation by B cells-mediated immune suppression through the overexpression of PD-1. Moreover, analysis of the relationship between PD-1 and inflammatory activity, further suggested that PD-1 was not only correlated with T cell immunity but also with other immune cells. These findings indicated that PD-1 plays essential immune and inflammatory functions in breast cancer. In our study, we focused on characterizing the role of PD-1; however, future studies should address whether there is any concomitant over-expression of PD-L1 on tumors, on immune cell populations, or on any of these immune cells in parallel with overexpression of PD-1. This would be helpful in gaining a deeper understanding of the association of PD1/PDL1 with related immune cell populations.

In summary, the expression of PD-1 was closely associated with the malignancy and might be as a potential biomaker in breast cancer, especially for TNBC. PD-1 might manipulate the anti-tumor immune response by impacting multiple immune cells, and this could vary with different tumors. Furthermore, PD-1 might synergize with other immune checkpoint members to modulate the immune microenvironment in breast cancer, which could be applied in the development of new targeted drugs for immunotherapy.

This study had limitations. Because detailed treatment information was not available in the TCGA database, treatment effects were potential confounders that should be considered when available and adjusted for appropriately. In certain cases, a proxy for standard of care, such as age, treating hospital, and year of diagnosis can alleviate some of the bias when treatment is unknown. A future study using an alterative source of data should address this problem.

## Data Availability Statement

Publicly available datasets were analyzed in this study. This data can be found here—TCGA: https://portal.gdc.cancer.gov/; METABRIC: http://www.cbioportal.org.

## Author Contributions

JW: Conception and design and study supervision. YF and ZW: Acquisition of data and development of methodology. QL and RC: Analysis and interpretation of data and writing the original manuscript. XK: Verification and revision of the manuscript. All authors contributed to the article and approved the submitted version.

## Funding

This work was supported by the Natural Science Foundation of China (No. 81872160); the Beijing Municipal Natural Science Foundation (Key Project) (No. 7191009); the Beijing Municipal Natural Science Foundation (No. 7204293); the Special Research Fund for Central Universities, Peking Union Medical College (No. 3332019053); the Beijing Hope Run Special Fund of Cancer Foundation of China (No. LC2019B03); the Beijing Hope Run Special Fund of Cancer Foundation of China (No. LC2019L07); the Golden Bridge Project Seed Fund of Beijing Association for Science and Technology; the PhD. Innovation Fund of Cancer Hospital, Chinese Academy of Medical Sciences (No. C2019-1051-09).

## Conflict of Interest

The authors declare that the research was conducted in the absence of any commercial or financial relationships that could be construed as a potential conflict of interest.

## Supplementary Material

Supplementary Figure 1 PD-1-related immune responses. Most of immune-related genes are positively correlated with PD-1 expression in TCGA and METABRIC databases, while a small number of genes are negatively associated (A and B), Subtypes denotes breast cancer molecular subtypes including Basal, basal-like; Her2, Her2-enriched; LumA, luminal A; LumB, luminal B.

Supplementary Figure 2 PD-1 expression is correlated with immunoinhibitors in pan-cancer.

Supplementary Figure 3 PD-1 expression is correlated with immunostimulators in pan-cancer.

Supplementary Figure 4 PD-1 expression is correlated with MHC molecules in pan-cancer.
