# Supplementary figures and images for: Molecular and Clinical Characterization of PD-1 in Breast Cancer Using Large-Scale Transcriptome Data
Source: Front Immunol. 2020 Nov 17;11:558757. doi: 10.3389/fimmu.2020.558757 (PMC7718028; doi:10.3389/fimmu.2020.558757)

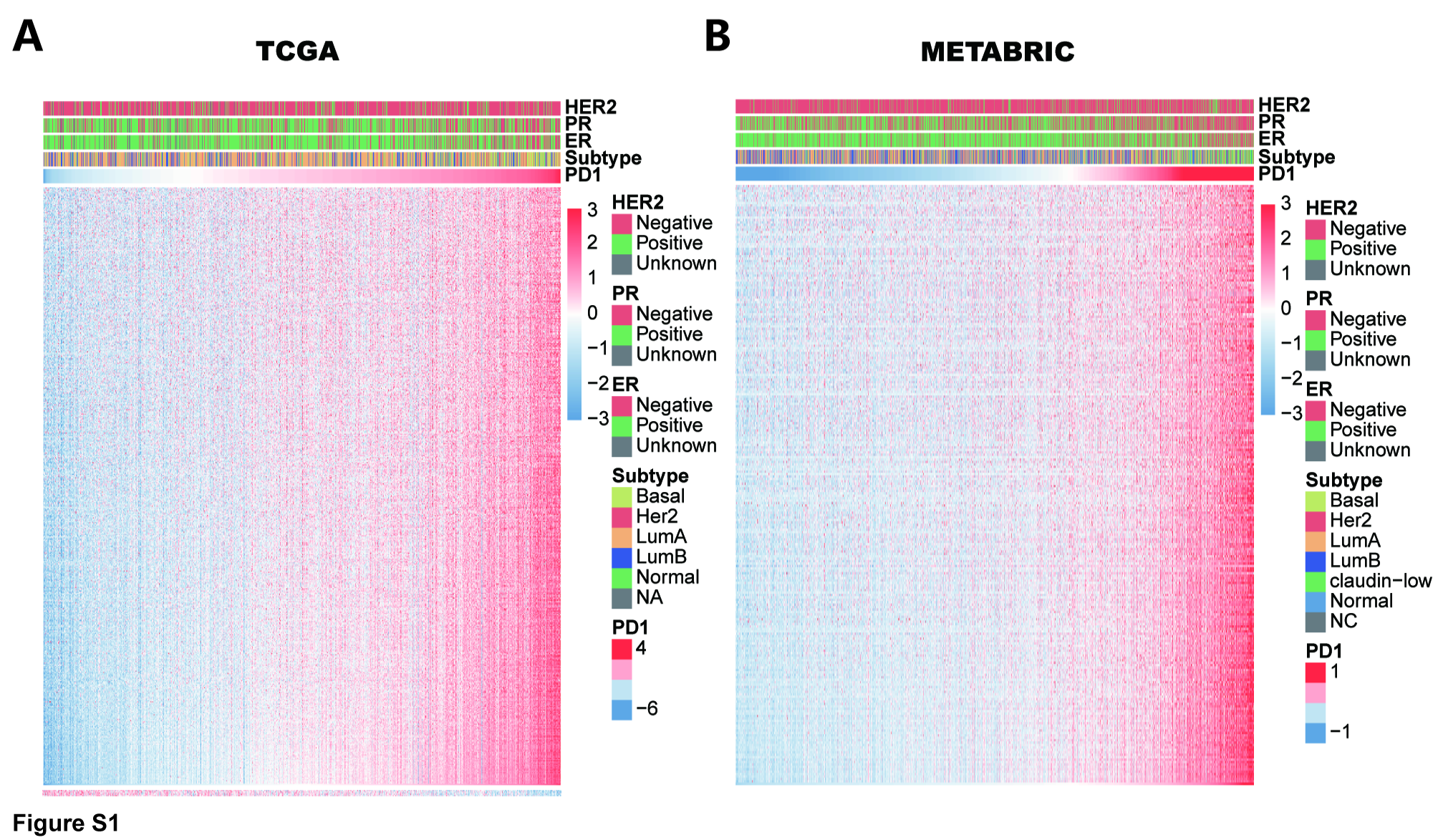

Supplement: Supplementary Figure 1 — PD-1-related immune responses. Most of immune-related genes are positively correlated with PD-1 expression in TCGA and METABRIC databases, while a small number of genes are negatively associated (A and B), Subtypes denotes breast cancer molecular subtypes including Basal, basal-like; Her2, Her2-enriched; LumA, luminal A; LumB, luminal B. [file Image_1.tif]

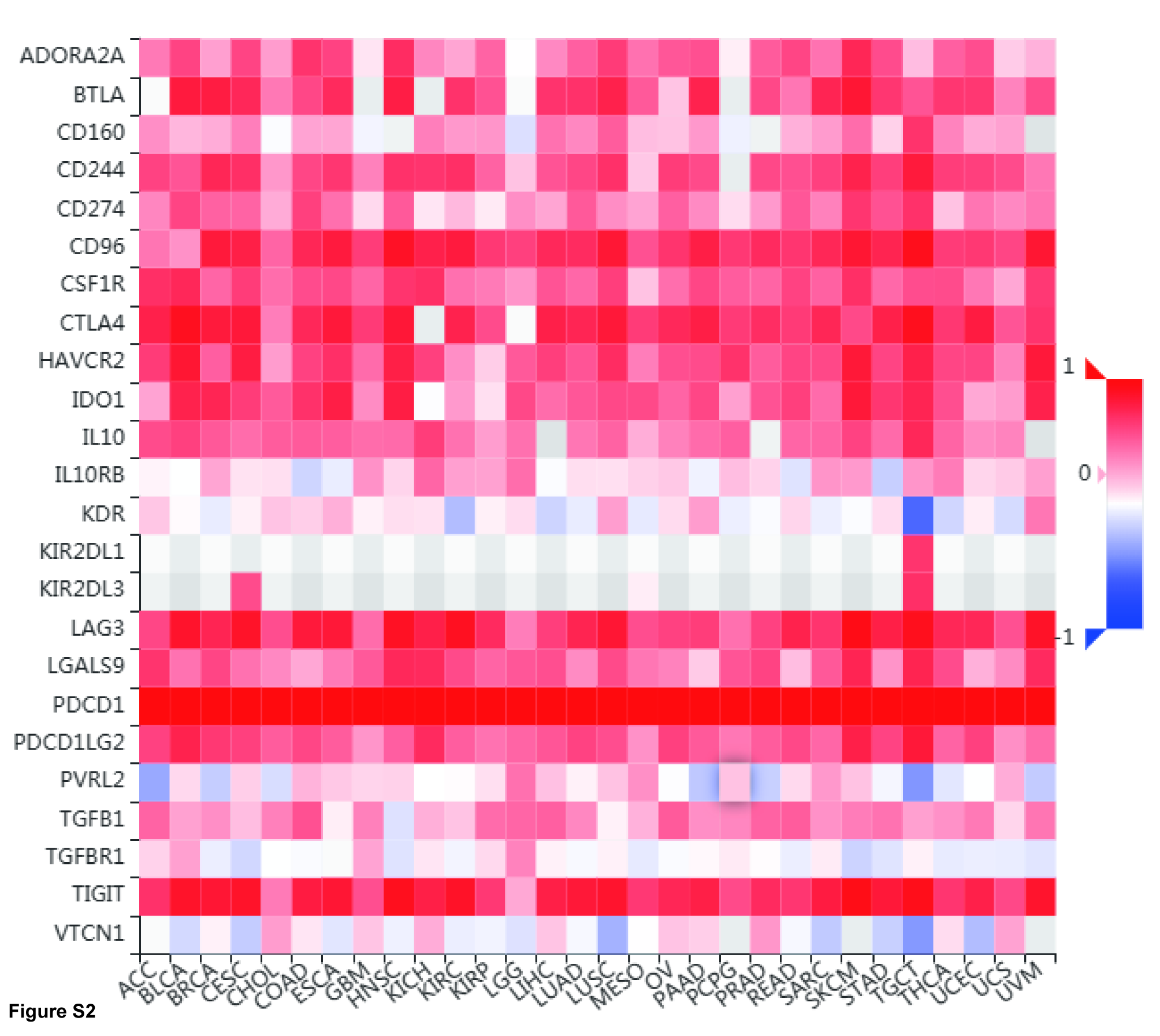

Supplement: Supplementary Figure 2 — PD-1 expression is correlated with immunoinhibitors in pan-cancer. [file Image_2.tif]

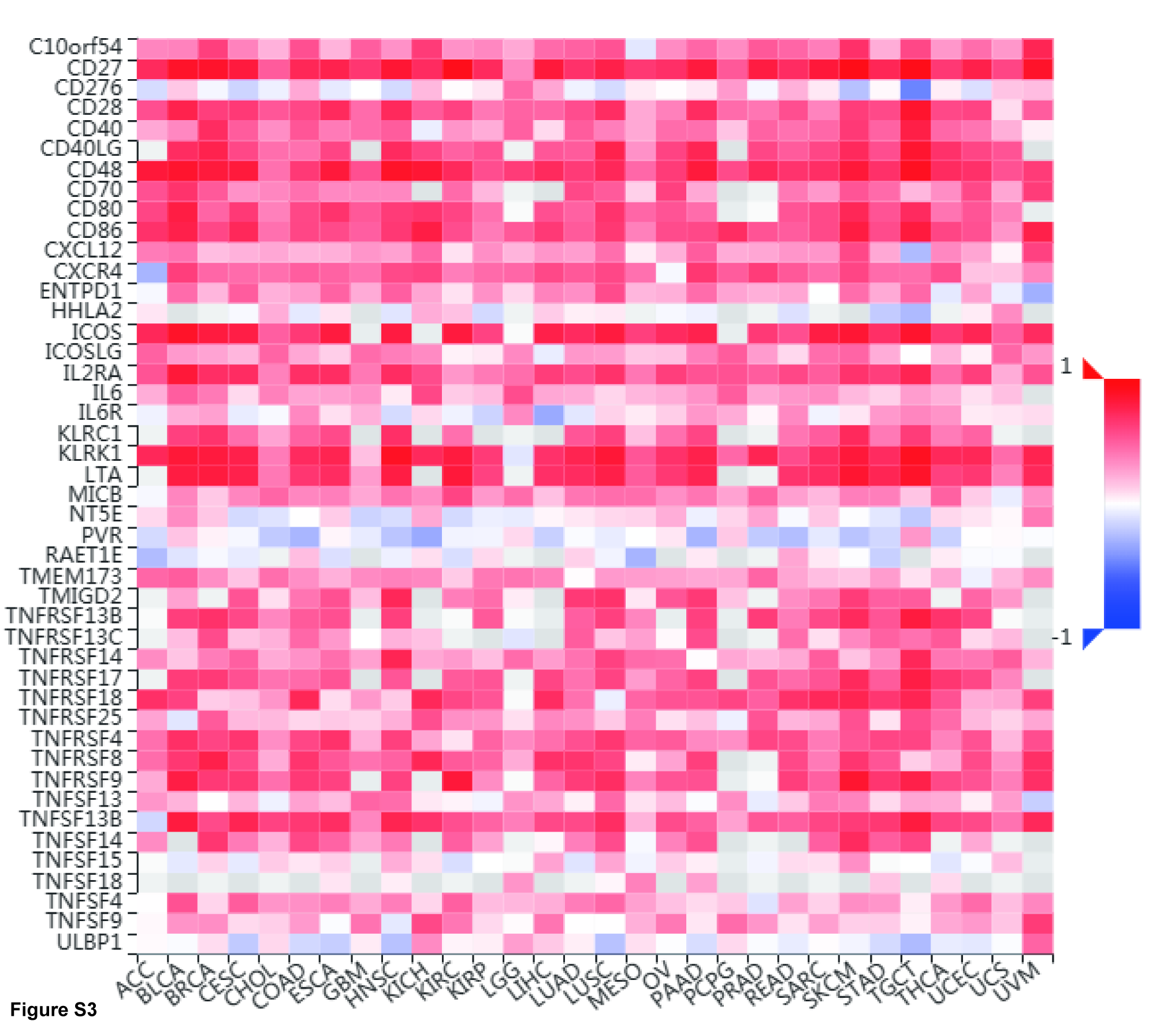

Supplement: Supplementary Figure 3 — PD-1 expression is correlated with immunostimulators in pan-cancer. [file Image_3.tif]

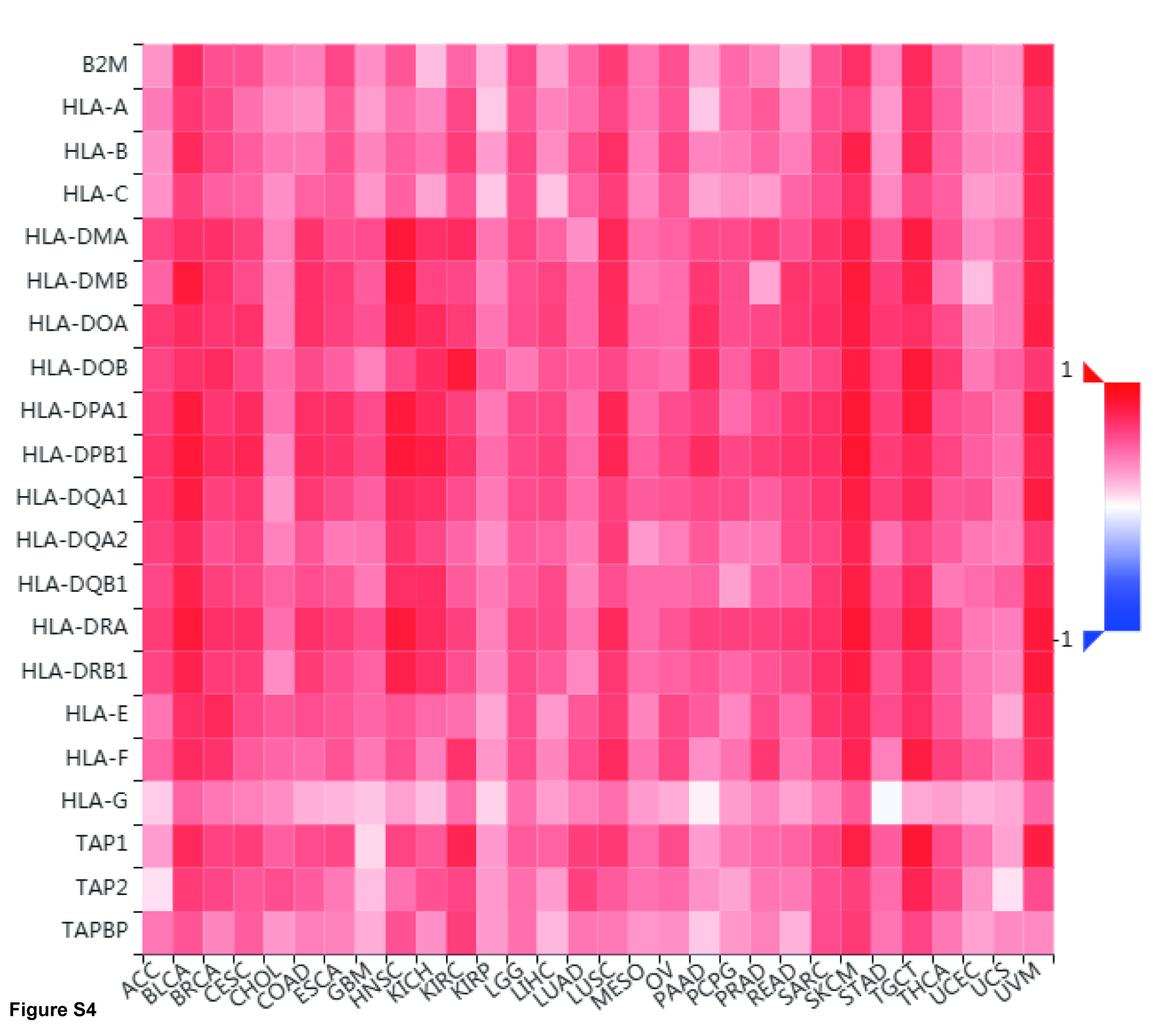

Supplement: Supplementary Figure 4 — PD-1 expression is correlated with MHC molecules in pan-cancer. [file Image_4.tif]
