# Supplementary material for: Molecular and Clinical Characterization of PD-1 in Breast Cancer Using Large-Scale Transcriptome Data
Source: Front Immunol. 2020 Nov 17;11:558757. doi: 10.3389/fimmu.2020.558757 (PMC7718028; doi:10.3389/fimmu.2020.558757)
Supplement: Supplementary file 5 [file Table_1.docx]

**Table S1. Clinical characteristics of patients in TCGA cohort**

|  | **Overall (n=1090)** |
| --- | --- |
| **Subtype** |  |
| **Basal** | 190 (17.4%) |
| Her2 | 82 (7.5%) |
| LumA | 563 (51.7%) |
| LumB | 215 (19.7%) |
| Normal | 40 (3.7%) |
| **Age (years)** |  |
| Mean (SD) | 58.5 (13.2) |
| Median [Min, Max] | 58.0 [26.0, 90.0] |
| **T** |  |
| 1 | 279 (25.6%) |
| 2 | 631 (57.9%) |
| 3 | 137 (12.6%) |
| 4 | 40 (3.7%) |
| Unknown | 3 (0.3%) |
| **N** |  |
| 0 | 514 (47.2%) |
| 1 | 360 (33.0%) |
| 2 | 120 (11.0%) |
| 3 | 76 (7.0%) |
| Unknown | 20 (1.8%) |
| **M** |  |
| M0 | 907 (83.2%) |
| M1 | 22 (2.0%) |
| Unknown | 161 (14.8%) |
| **AJCC stage** |  |
| 1 | 181 (16.6%) |
| 2 | 621 (57.0%) |
| 3 | 250 (22.9%) |
| 4 | 20 (1.8%) |
| Unknown | 18 (1.7%) |
| **ER** |  |
| Negative | 236 (21.7%) |
| Positive | 803 (73.7%) |
| Unknown | 51 (4.7%) |
| **PR** |  |
| Negative | 343 (31.5%) |
| Positive | 694 (63.7%) |
| Unknown | 53 (4.9%) |
| **HER2** |  |
| Negative | 895 (82.1%) |
| Positive | 168 (15.4%) |
| Unknown | 27 (2.5%) |
| **TNBC** |  |
| Non-TNBC | 863 (79.2%) |
| TNBC | 177 (16.2%) |
| Unknown | 50 (4.6%) |
| **gender** |  |
| FEMALE | 1078 (98.9%) |
| MALE | 12 (1.1%) |
