# Supplementary material for: Molecular and Clinical Characterization of PD-1 in Breast Cancer Using Large-Scale Transcriptome Data
Source: Front Immunol. 2020 Nov 17;11:558757. doi: 10.3389/fimmu.2020.558757 (PMC7718028; doi:10.3389/fimmu.2020.558757)
Supplement: Supplementary file 6 [file Table_2.docx]

**Table S2. Clinical characteristics of patients in METABRIC cohort**

|  | **Overall (n=1904)** |
| --- | --- |
| **Subtype** |  |
| Basal | 199 (10.5%) |
| claudin-low | 199 (10.5%) |
| Her2 | 220 (11.6%) |
| LumA | 679 (35.7%) |
| LumB | 461 (24.2%) |
| NC | 6 (0.3%) |
| Normal | 140 (7.4%) |
| **Age (years)** |  |
| Mean (SD) | 61.1 (13.0) |
| Median [Min, Max] | 61.8 [21.9, 96.3] |
| **ER** |  |
| Negative | 445 (23.4%) |
| Positive | 1459 (76.6%) |
| **PR** |  |
| Negative | 895 (47.0%) |
| Positive | 1009 (53.0%) |
| **HER2** |  |
| Negative | 1668 (87.6%) |
| Positive | 236 (12.4%) |
| **AJCC stage** |  |
| 0 | 4 (0.2%) |
| 1 | 475 (24.9%) |
| 2 | 800 (42.0%) |
| 3 | 115 (6.0%) |
| 4 | 9 (0.5%) |
| Unknown | 501 (26.3%) |
| **GRADE** |  |
| 1 | 165 (8.7%) |
| 2 | 740 (38.9%) |
| 3 | 927 (48.7%) |
| Unknown | 72 (3.8%) |
| **BREAST SURGERY** |  |
| BREAST CONSERVING | 755 (39.7%) |
| MASTECTOMY | 1127 (59.2%) |
| Missing | 22 (1.2%) |
| **HORMONE THERAPY** |  |
| NO | 730 (38.3%) |
| YES | 1174 (61.7%) |
| **CHEMOTHERAPY** |  |
| NO | 1508 (79.2%) |
| YES | 396 (20.8%) |
