# Supplementary material for: Molecular and Clinical Characterization of PD-1 in Breast Cancer Using Large-Scale Transcriptome Data
Source: Front Immunol. 2020 Nov 17;11:558757. doi: 10.3389/fimmu.2020.558757 (PMC7718028; doi:10.3389/fimmu.2020.558757)
Supplement: Supplementary file 7 [file Table_3.docx]

| **Table S3 Detailed R and p-value of correlation between PD-1 and other checkpoint members** | | | | | | | | |
| --- | --- | --- | --- | --- | --- | --- | --- | --- |
| **TCGA** | | | | | **METABRIC** | | | |
| **Gene_1** | **Gene_2** | **Correlation** | **P-value** | **Gene_1** | | **Gene_2** | **Correlation** | **P-value** |
| PDCD1 | PDCD1LG2 | 0.649 | P<0.001 | PDCD1 | | PDCD1LG2 | 0.285 | P<0.001 |
| PDCD1 | CD274 | 0.592 | P<0.001 | PDCD1 | | CD274 | 0.317 | P<0.001 |
| PDCD1 | CTLA4 | 0.821 | P<0.001 | PDCD1 | | CTLA4 | 0.702 | P<0.001 |
| PDCD1 | IDO1 | 0.755 | P<0.001 | PDCD1 | | IDO1 | 0.639 | P<0.001 |
| PDCD1 | LAG3 | 0.75 | P<0.001 | PDCD1 | | LAG3 | 0.642 | P<0.001 |
| PDCD1 | BTLA | 0.808 | P<0.001 | PDCD1 | | BTLA | 0.599 | P<0.001 |
| PDCD1 | ICOS | 0.81 | P<0.001 | PDCD1 | | ICOS | 0.713 | P<0.001 |
| PDCD1 | CD27 | 0.862 | P<0.001 | PDCD1 | | CD27 | 0.699 | P<0.001 |
| PDCD1 | CD40 | 0.718 | P<0.001 | PDCD1 | | CD40 | 0.556 | P<0.001 |
| PDCD1 | CD48 | 0.817 | P<0.001 | PDCD1 | | CD48 | 0.627 | P<0.001 |
